# A Mucosal Subunit Vaccine Protects against Lethal Respiratory Infection with *Francisella tularensis* LVS

**DOI:** 10.1371/journal.pone.0050460

**Published:** 2012-11-28

**Authors:** Amit R. Ashtekar, Jannet Katz, Qingan Xu, Suzanne M. Michalek

**Affiliations:** 1 Department of Microbiology, University of Alabama at Birmingham, Birmingham, Alabama, United States of America; 2 Department of Pediatric Dentistry, University of Alabama at Birmingham, Birmingham, Alabama, United States of America; Albany Medical College, United States of America

## Abstract

*Francisella tularensis* (FT) is a highly virulent pathogen for humans and other mammals. Severe morbidity and mortality is associated with respiratory FT infection and there are concerns about intentional dissemination of this organism. Therefore, FT has been designated a category A biothreat agent and there is a growing interest in the development of a protective vaccine. In the present study, we determine the protective potential of a subunit vaccine comprised of the FT heat shock protein DnaK and surface lipoprotein Tul4 against respiratory infection with the live vaccine strain (LVS) of FT in mice. First, we establish an optimal intranasal immunization regimen in C57BL/6 mice using recombinant DnaK or Tul4 together with the adjuvant GPI-0100. The individual immunization regimens induced robust salivary IgA, and vaginal and bronchoalveolar IgA and IgG antigen-specific antibodies. Serum IgG1 and IgG2c antibody responses were also induced, indicative of a mixed type 2 and type 1 response, respectively. Next, we show that immunization with DnaK and Tul4 induces mucosal and systemic antibody responses that are comparable to that seen following immunization with each antigen alone. This immunization regimen also induced IFN-γ, IL-10 and IL-17A production by splenic CD4^+^ T cells in an antigen-specific manner. Importantly, over 80% of the mice immunized with DnaK and Tul4, but not with each antigen alone, were protected against a lethal respiratory challenge with FT LVS. Protection correlated with reduced bacterial burden in the lung, liver and spleen of mice. This study demonstrates the potential of DnaK and Tul4 as protective antigens and lends support to the notion of combining distinct, immunodominant antigens into an effective multivalent tularemia vaccine.

## Introduction

*Francisella tularensis* (FT) is a facultative, intracellular, Gram-negative coccobacillus and the causative agent of tularemia, a zoonotic disease. Humans can acquire infection by bites from ticks or mosquitoes, handling carcasses of infected wildlife, drinking contaminated water or inhaling infectious aerosols [1], [2]. Among the various types of tularemia, respiratory tularemia is a major health concern, since failure to initiate prompt antibiotic treatment can lead to high mortality rates [1], [2]. Considering the extreme virulence, the ability to persist for weeks in nature, and the probability of being intentionally disseminated, the Centers for Disease Control and Prevention has categorized FT subspecies *tularensis* (type A, Schu S4) as a category A biological agent [1]. Lack of an effective vaccine and the current threat of biological misuse of this organism have led to a renewed interest in the development of protective vaccines against FT infection.

The type B strain (FT subspecies *holarctica*) causes moderate disease in humans and was used for the development of an attenuated live vaccine strain (LVS). However, FT LVS is not licensed for the general population since the attenuating mutations have not been fully characterized. Although FT LVS is attenuated in humans, it is highly virulent in mice, causing a disease that closely resembles human tularemia [3]. Therefore, FT LVS infection in mice has been extensively used as an initial experimental approach to test potential vaccine candidates and suggest possible vaccination strategies against the more virulent type A strain.

When cases of tularemia were reported at Martha’s Vineyard, MA, 11 out of 15 cases were primarily pneumonic [4]. It can also be presumed that if intentionally disseminated, FT infection will most likely occur via mucosal surfaces [5]. Therefore, a developmental vaccine against FT infection needs to induce protective responses at mucosal surfaces as a first line of defense. Additionally, since FT has an extracellular phase in the blood and can disseminate in the host [2], [6], it is important that the potential vaccine also induces protective immune responses in the systemic compartment. While vaccines injected via a parenteral route lead to strong systemic immunity, they are generally poor inducers of mucosal immunity. However, immunization via a mucosal route can induce both mucosal and systemic immune responses [7]. Therefore, the development of a mucosal vaccine is likely a more preferred way to induce protection against FT infection. Furthermore, in addition to antibodies, a vaccine against FT, should also elicit cellular immune responses [8]. Antibodies and cellular immune responses can synergize to better combat FT infection [9].

The selection of the appropriate adjuvant as part of a vaccine is critical since the characteristics of an induced response may be influenced by the adjuvant used [10]–[12]. That is, the balance of stimulated Th1/Th2 cells, subsequent cytokine production and the resulting IgG subclass response to the antigen can be affected by an adjuvant. Among the adjuvants shown to be stable, lowly toxic and capable of stimulating antibody and cell-mediated immune responses is GPI-0100 (GPI), a semi-synthetic adjuvant derived by modifying saponins extracted from the bark of the South American tree Quillaja saponaria Molina [13]. GPI was developed to retain the adjuvant properties of quillaja saponins, but to exhibit less toxicity and greater stability in aqueous solution. GPI is believed to enhance immune responses to exogenous antigens by having a stimulatory effect on antigen-presenting cells (APC), as well as T cells [14]. Furthermore, previous studies from our laboratory have demonstrated the ability of GPI to potentiate mucosal, as well as systemic antibody responses to a bacterial protein [15].

Several approaches are currently being pursued to develop a protective vaccine against tularemia, including deriving genetically defined attenuated mutant strains, employing inactivated organisms and identifying immunodominant antigens for their potential use in subunit vaccines [16]. Among these approaches, subunit vaccines are considered safer because they can be precisely defined to eliminate undesirable properties of a complex microbial vaccine. Various FT antigens have the potential for use in a subunit vaccine, including lipopolysaccharide (LPS), outer membrane proteins (OMPs) and intracellular heat shock proteins (HSPs). Thus far, most studies have employed FT LPS and have shown that it has a significant protective potential against tularemia, thus making it a desirable candidate for a subunit vaccine [17]–[20]. However, only a handful of studies have assessed the potential of individual OMPs [21]–[24] and intracellular HSPs as subunit vaccine candidates [25]–[28]. Moreover, attempts have not been made to combine distinct, immunodominant protein/lipoprotein antigens into a potential multivalent vaccine and assess its effectiveness in conferring protection against tularemia.

In this study, we demonstrate that a subunit vaccine comprising of the heat shock protein DnaK and the surface lipoprotein Tul4 protected mice against a lethal respiratory infection with FT LVS. These results demonstrate the protective potential of DnaK and Tul4, and support the concept of combining immunodominant antigens for the development of an effective multivalent tularemia vaccine.

## Materials and Methods

### Ethics Statement

All studies were done in accordance with the recommendations of the Guide for the Care and Use of Laboratory Animals of the National Institute of Health. All protocols involving animal research were approved by the Institutional Animal Care and Use Committee of the University of Alabama at Birmingham (UAB; Protocol number 09112 under Institutional Animal Assurance Number A-3255-01).

### Mice

C57BL/6 wild-type mice were bred and maintained within an environmentally controlled, pathogen-free animal facility at UAB. Female mice, 8 to 10 weeks of age, were used in all the experiments.

### Bacteria

FT LVS (ATCC 29684; American Type Culture Collection, Rockville, MD), kindly provided by Karen Elkins (Division of Bacterial and Parasitic Products, CBER/FDA, Bethesda, MD), was grown as described previously with certain modifications [29]. Initially, the original frozen stock of FT LVS was streaked on cysteine heart agar (CHA) plates supplemented with 5% defibrinated sheep blood (I-Tek Medical Tech. St. Paul, MN). After incubation for 3–4 days at 37°C in 5% CO_2_ and at 95% humidity, a single isolated colony was picked and used to inoculate Mueller-Hinton II (MH-II) broth (BD Biosciences, Sparks, MD) (50 ml) supplemented with 2% IsoVitaleX Enrichment (BD Biosciences), 0.1% glucose, 63 mM CaCl_2_, 53 mM MgCl_2_, and 34 mM ferric pyrophosphate. The inoculated broth cultures were incubated at 37°C with shaking (180 rpm), and at mid-log phase (O.D. 600 nm <0.3) the bacteria were collected by centrifugation. The bacterial pellet was suspended in 25 ml sterile phosphate buffered saline (PBS) supplemented with 1.3% gelatin, aliquoted and stored at −80°C. The number of bacteria in the final suspension were 10^9^ CFU/ml as determined by plating serial dilutions on MH-II agar plates supplemented with 1% bovine hemoglobin (BD Biosciences) and 2% IsoVitaleX Enrichment (BD Biosciences).

### Antigens and Adjuvant

The chromatographically fractionated quillaja saponin derivative GPI was obtained from Galenica Pharmaceuticals, Inc. (Birmingham, AL). FT DnaK and Tul4 were expressed in a bacterial expression system and purified as previously described [30], [31]. Briefly, the gene encoding DnaK (originally obtained from Anders Sjostedt, Umea University, Sweden) was cloned into the pET-23d vector (Novagen, Madison, WI) and was used to transform *E. coli* strain BL21 (DE3) pLysS for protein expression (Novagen, Madison, WI). Protein expression was induced following the addition of 0.5 mM isopropyl β-D-thiogalactoside (IPTG) for 4 h. DnaK was purified using a three-step purification procedure comprised of affinity, anion exchange and size exclusion chromatography, and has been shown to be free of endotoxin (LPS) [30]. The *E. coli* strain expressing Tul4 was kindly provided by Fabio Re (University of Tennessee Health Science Center, Memphis, TN) and Tul4 was purified as described previously [31], with some modifications. Briefly, the gene encoding Tul4 was cloned into the pET-28a vector (Novagen) and used to transform the *E. coli* BL21 (DE3) lpxM strain. Tul4 expression was induced for 4 h using 0.1 mM IPTG. The bacteria were harvested by centrifugation and the pellet was suspended in cold PBS supplemented with 350 mM NaCl, 2% Triton X-114 (PTX) containing protease inhibitor cocktail tablets (Complete, Mini, EDTA-free, Roche Applied Science, Indianapolis, IN). To aid cell lysis, bacteria were sonicated for 3–5 min using a Sonic Dismembranator model 500 (Fisher Scientific, Pittsburgh, PA) with a temperature probe that maintained the temperature below 16°C. Cell debris was cleared by centrifugation and the supernatant was incubated at 37°C to induce detergent phase separation. After centrifugation at 14,000 rpm for 25 min at room temperature, the upper aqueous phase was discarded and replaced with a similar volume of PBS supplemented with 350 mM NaCl. The procedure of phase separation was repeated twice, and the final detergent phase was resuspended in ice cold PBS supplemented with 350 mM NaCl to the original volume. The sample was filtered through a 0.22 µm filter before applying to a HisPrep Nickel column (Amersham Biosciences/GE Healthcare, Piscataway, NJ). The column was washed with 6–8 column volumes of cold PTX and the bound Tul4 was then eluted using a gradual imidazole gradient (10–300 mM). Eluted fractions containing purified Tul4 were pooled and sterilized by using a 0.22 µm filter. The detergent was then removed by precipitation by adding ∼2.5 volumes of ethanol and incubated for 48 h at −20°C. After centrifugation, the pellet was air-dried and resuspended in sterile PBS supplemented with 350 mM NaCl.

### Immunogenic Potential of DnaK and Tul4 Following FT LVS Infection

The in vivo immunogenicity study was done as previously described [32]. Briefly, non-anesthetized C57BL/6 mice were inoculated with FT LVS (2×10^5^ CFU) via the intranasal (i.n.) route and spleens were harvested on day 7 to examine the acute response post-inoculation. Spleen cells from infected mice were cultured (2.5×10^6^/ml) in RPMI-1640 culture medium (Cellgro Mediatech, Washington, DC) supplemented with 10% fetal bovine serum (FBS), 2 mM L-glutamine, 50 µM 2-mercaptoethanol, 20 mM HEPES, 1 mM sodium pyruvate, 1.5 mg/ml of sodium bicarbonate, 50 U/ml penicillin, and 50 µg/ml streptomycin (RPMI-1640 complete medium). Cells were stimulated for 18–24 h with FT LVS extract (FT extract; 100 µg/ml), DnaK (20 µg/ml), Tul4 (1 µg/ml) or an unrelated *Streptococcus mutans* saliva-binding region protein (SBR; 20 µg/ml). FT extract was prepared by placing FT LVS in 70% ethanol, as previously described [33]. Brefeldin A (GolgiPlug, BD Biosciences) was added to cultures for the last 4 h of incubation. Cells were then washed and surface stained with phycoerythrin (PE) labeled anti-CD4 or anti-CD8 antibody (eBioscience, San Diego, CA), followed by fixing and permeablizing according to the manufacturer’s instructions (eBioscience). Cells were then stained with allophycocyanin-conjugated antibody to IFN-γ. Data were collected using a FACScalibur flow cytometer and analyzed with the CellQuest software (BD Biosciences, San Jose, CA).

### Intranasal Immunizations with DnaK and Tul4

Mice were immunized by the i.n. route with DnaK (10 or 20 µg) alone or with GPI (100 µg) on days 0 and 14 or on days 0, 14 and 28, or with Tul4 (1 or 10 µg) alone or with GPI (100 µg) on days 0, 14 and 28. Additional groups of mice were immunized with DnaK (20 µg)+Tul4 (10 µg)+GPI (100 µg) on days 0, 14 and 28. Control mice received PBS or GPI only. In all cases, the antigens and adjuvant were combined into a final volume of 25 µl and 12.5 µl was slowly instilled in each nare of non-anesthetized mice with a 10 min interval between the 2 applications.

### Collection of Samples

Serum, saliva and vaginal wash samples were collected prior to immunization and at approximately 2-week intervals following the initial immunization. Blood samples were collected from the retro-orbital plexus of mice anesthetized with isoflurane using heparinized capillary tubes and the serum was obtained after centrifugation. Saliva samples (∼100 µl) were collected over a 20 min interval after stimulation of the saliva flow by intraperitoneal (i.p.) injection of carbachol (5 µg in 0.1 ml/mouse; Sigma, St. Louis, MO), as previously described [15]. Vaginal wash samples were collected at room temperature by flushing the vagina twice, with 50 µl of PBS. For assessment of antibody responses in the lungs, additional groups of mice were immunized on days 0, 14 and 28, as described above and bronchoalveolar lavage (BAL) samples were collected two weeks after the last immunization, as previously described [34]. All samples were stored at −20°C until assayed for antibody activity.

### Antibody Measurement

Serum, saliva, vaginal wash and BAL samples were assessed for antibody activity to DnaK or Tul4 by ELISA, as previously described [15]. Briefly, microtiter plates (NUNC International, Roskilde, Denmark) were coated with purified DnaK or Tul4 (1 µg/ml) or with goat anti-mouse IgA, IgG, IgG1 or IgG2c antibodies (0.50 µg/ml; Southern Biotechnology Associates, Inc., Birmingham, AL) in borate buffered saline (BBS). Blocking was done for 4 h at room temperature with BBS containing 1% bovine serum albumin (BSA). Serial twofold dilutions of the samples were added to wells in duplicate and the plates were incubated overnight at 4°C. All dilutions were made in BBS containing 1% BSA. Samples were developed by the addition of the appropriate HRP-conjugated goat anti-mouse IgG, IgG1, IgG2c or IgA antibody (Southern Biotechnology), followed by *o*-phenylenediamine substrate (Sigma) with H_2_O_2_. The concentration of antibodies in the samples was determined by interpolation on standard curves generated using a mouse immunoglobulin reference serum (ICN Biomedical, Inc., Costa Mesa, CA) and constructed by a computer program based on four parameter logistic algorithms (Softmax/Molecular Devices Corp., Menlo Park, CA).

### Total and CD4^+^ T Cell Cultures

Groups of mice were immunized with GPI (100 µg) alone or with DnaK (20 µg)+Tul4 (10 µg)+GPI (100 µg) on days 0, 14 and 28 via the i.n. route. Seven days after the last immunization, spleens were isolated, processed into single cell suspensions and a portion of the cells were cultured (2.5×10^6^/ml) in tissue culture plates containing RPMI-1640 complete medium in a humidified 5% CO_2_ incubator at 37°C. Spleen cells were stimulated for 4 days with various concentrations of DnaK and/or Tul4, and supernatants were harvested and assessed for IFN-γ (eBioscience, San Diego, CA), IL-10 and IL-17A cytokines (R&D Systems, Minneapolis, MN) by ELISA, according to the manufacturer’s instructions.

The remaining spleen cell suspensions from the immunized mice were used to purify CD4^+^ T cells by negative selection using a CD4^+^ T cell isolation kit (Miltenyi Biotec Inc, Auburn, CA), according to the manufacturer’s instructions. Purified CD4^+^ T cells were then cultured (2.5×10^6^/ml) alone in RPMI-1640 complete medium or co-cultured with irradiated splenocytes/APC (3000 rads) that were derived from naive wild-type mice at different APC:T cell ratios, as indicated in the respective figures. Cell cultures were stimulated for 4 days with various concentrations of DnaK and/or Tul4 and supernatants were harvested and assessed for IFN-γ, IL-10 and IL-17A by ELISA, as stated above.

### Mice Infection Model

Respiratory infection was performed by i.n. instillation of the bacterial inoculum. Prior to infection, a frozen bacterial stock of FT LVS was thawed and diluted in sterile PBS to the desired dose. Mice were anesthetized by i.p. injection of ketamine HCl (50–75 mg/kg) and xylazine (5–7.5 mg/kg), since this procedure facilitates delivery of the inoculum to the pulmonary compartment [35], [36]. Each mouse received 25 µl of PBS containing the indicated number of CFU via the i.n. route. The actual numbers of FT inoculated at the time of infection were confirmed by plating serial dilutions on MH-II agar plates. For assessment of protection, mice were monitored daily for mortality and morbidity.

### Bacterial Burden and Serum Cytokines

Relative levels of bacteria in the tissues of infected mice were assessed by quantifying the levels of FT LVS-specific 16s ribosomal (r)DNA, as previously described [37], [38] with modifications, and served as an indirect measure of bacterial burden. Total DNA was purified from livers, lungs and spleens by using a DNeasy Blood & Tissue Kit (Qiagen), according to the manufacturer’s instructions. One µg of total DNA was used in a real-time PCR system for amplification of FT LVS-specific 16s rDNA. The PCR primers used were as follows: forward (5′-CAGCCACATTGGGACTGAGA-3′) and reverse (5′-CACACATGGCATTGCTGGAT-3′). Real-time PCR was performed by using a Lightcycler (Roche Molecular Biochemicals, Indianapolis, IN) with a Lightcycler FastStart DNA Master SYBR Green I reagent (Roche Applied Science, Germany), according to the manufacturer's instructions. Samples were subjected to 45 cycles of amplification at 95°C for 10 s, followed by 59°C for 5 s and 72°C for 15 s. The relative amount of FT 16s rDNA in each sample was calculated based upon a standard curve generated by simultaneously amplifying serial ten-fold dilutions of genomic DNA isolated from FT LVS cultures.

Separate groups of immunized and infected mice were used for the collection of serum on days 2, 3 and every other day thereafter until the mice succumbed to infection. In surviving mice, serum was collected until day 15 post infection. Serum samples were stored frozen until assessed for various cytokines, chemokines, and growth factors using a mouse multiplex kit (Millipore, Billerica, MO). The following analytes were assessed; granulocyte colony-stimulating factor (G-CSF), IFN-γ, IL-1α, IL-6, IL-10, IL-12 p70, IL-17A, TNF-α, inflammatory protein-10 (IP-10), regulated on activation, normal T cell expressed and secreted (RANTES), and the CXC chemokine CXCL1 (KC).

### Statistical Analysis

Statistical significance for differences in antibody levels between immunized groups and cytokine levels in vitro cultures was evaluated by analysis of variance and the Tukey-Kramer multiple-comparisons test. Statistical significance for survival data was determined by analyzing the mean time to death by the Kruskal-Wallis test and the Dunn’s multiple-comparisons test. Bacterial burden data was compared using an unpaired t-test. InStat program (Graphpad Software, San Diego, CA) was used for all data analysis.

## Results

### Respiratory Infection with FT LVS Induces CD4^+^ and CD8^+^ T Cells Specific for DnaK and Tul4

**Figure 1:**
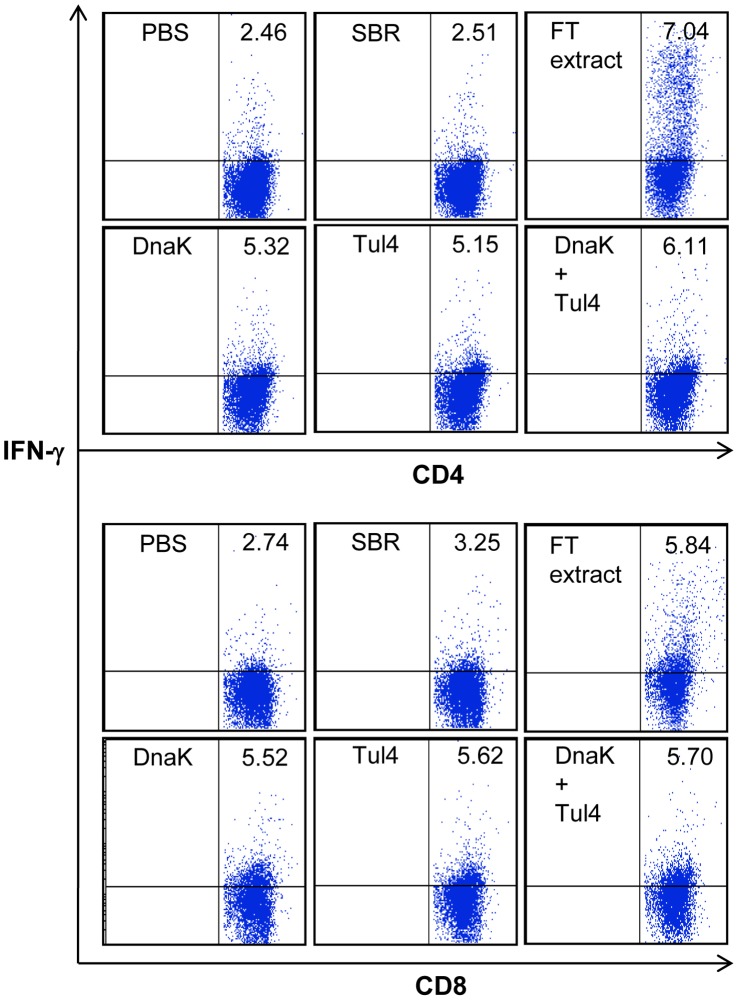
DnaK and Tul4 are recognized by CD4^+^ and CD8^+^ T cells during respiratory FT LVS infection. Spleen cells from FT LVS infected mice were cultured for 18–24 h with DnaK, Tul4, DnaK+Tul4 or with FT LVS extract (FT extract), which served as a positive control. Negative control wells were cultured with an irrelevant protein, SBR, or media alone. Protein transport inhibitor, brefeldin A, was added to cultures during the last 4 h of incubation, and then cells were stained with fluorochrome-labeled antibodies followed by FACS analysis. Representative FACS dot plots for intracellular IFN-γ positive CD4^+^ and CD8^+^ T cells are shown.

In our first series of studies, we determined whether DnaK and Tul4 are recognized by the host adaptive immune system during FT LVS infection. Splenic CD4^+^ and CD8^+^ T cells from mice infected via the i.n. route with FT LVS (7 days post-infection) responded to in vitro stimulation with purified DnaK and Tul4 by producing IFN-γ (Figure 1), demonstrating that DnaK and Tul4 are indeed processed and presented to T cells following infection with FT LVS. As expected, CD4^+^ and CD8^+^ T cells from FT LVS infected mice did not respond to the unrelated SBR protein. Whereas, the FT extract stimulated the highest CD4^+^ and CD8^+^ T cell responses, most probably because the extract contains several immune epitopes capable of inducing a response by the T cells. This data demonstrates that DnaK and Tul4 contain epitopes that induce a cell-mediated response following FT LVS infection in vivo, and thus may serve as protective antigens.

### Immunogenicity of DnaK

**Figure 2:**
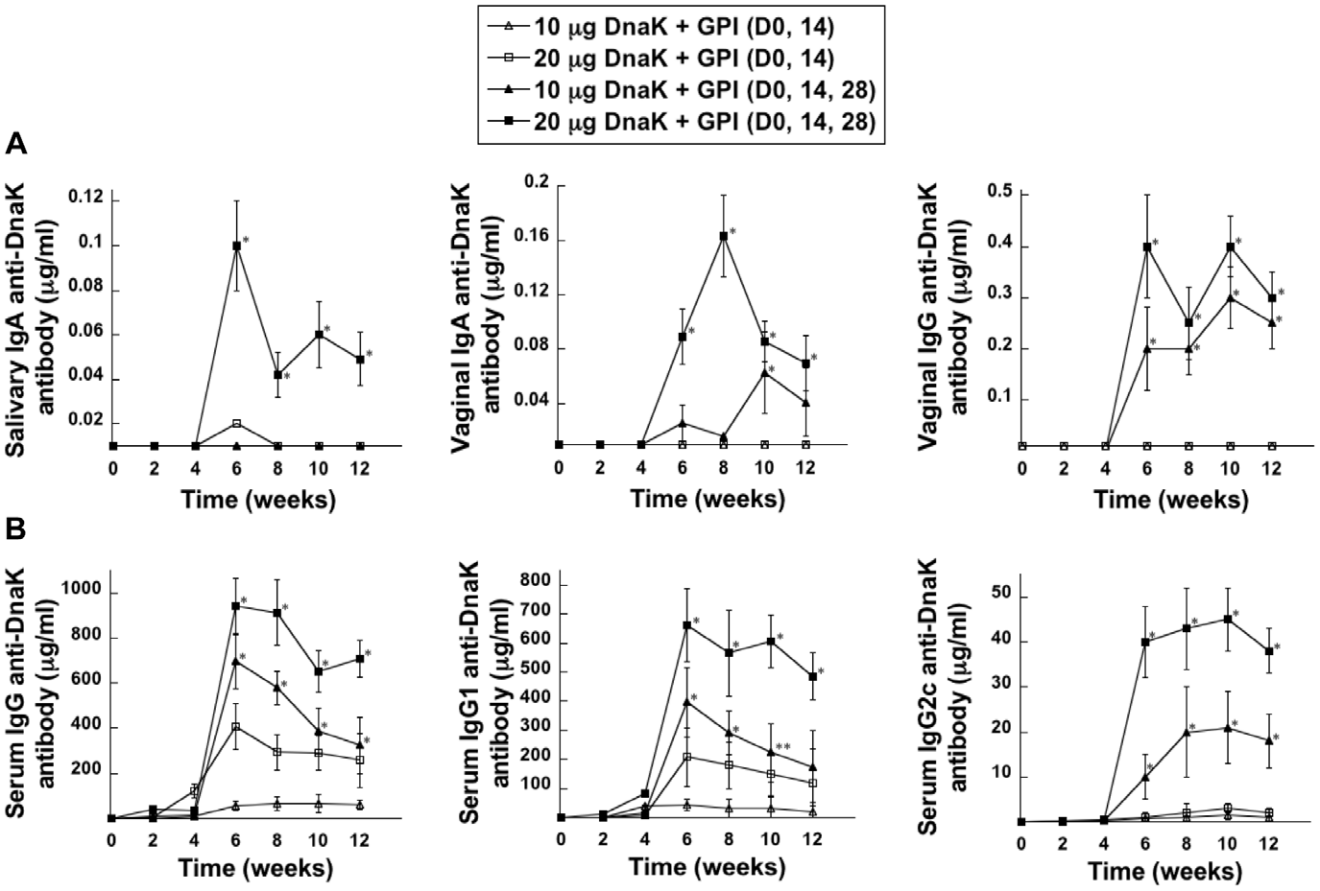
DnaK-specific mucosal and serum antibody responses following i.n. immunization with DnaK+GPI. Mice were immunized via the i.n. route with DnaK (10 or 20 µg)+GPI (100 µg) on days 0 and 14 or on days 0, 14 and 28. Individual saliva, vaginal wash and serum samples were collected prior to and at approximately 2-week intervals following immunization. Levels of DnaK-specific IgA in the saliva and vaginal wash samples and IgG in the vaginal wash samples (A), and of IgG, IgG1 and IgG2c in the serum (B), were determined by ELISA. There were 6 mice per experimental group and values are expressed as the mean ± SEM. Significant differences were seen at *P*<0.001 (*) and *P*<0.01 (**) compared with mice receiving only two immunizations with equivalent doses of DnaK and GPI.

In order to determine the optimal dose and immunization regimen for the induction of mucosal and systemic antibody responses to DnaK following mucosal immunization, groups of mice were immunized via the i.n. route with DnaK alone (10 or 20 µg) or with the adjuvant GPI (100 µg) on days 0 and 14 or on days 0, 14 and 28. Immunization with DnaK alone on days 0 and 14 or on days 0, 14 and 28 did not induce detectable salivary or vaginal antibody responses (data not shown). When DnaK was used in conjunction with the adjuvant GPI, two immunizations induced minimal salivary IgA and undetectable vaginal IgA and IgG anti-DnaK antibody responses (Figure 2A). However, mice receiving a third dose of DnaK+GPI exhibited robust and significantly higher salivary IgA and vaginal IgA and IgG anti-DnaK antibody levels compared to mice receiving 2 immunizations. These results demonstrate that three immunizations are required to induce DnaK-specific salivary and vaginal antibody responses. DnaK at 20 µg was most effective in inducing salivary IgA anti-DnaK antibodies, and a dose-dependent increase in vaginal IgA and IgG anti-DnaK antibody responses was observed when the immunizing dose of DnaK was increased from 10 µg to 20 µg. The anti-DnaK response peaked between 2–4 weeks after the third immunization, and persisted for at least 8 weeks after the last immunization (until week 12).

Although a third immunization was essential to induce antibody responses to DnaK in the mucosal compartment, serum IgG, IgG1 and slight IgG2c anti-DnaK antibody responses were induced following a second immunization with DnaK+GPI (Figure 2B). However, mice receiving a third immunization had significantly higher serum IgG, IgG1 and IgG2c anti-DnaK antibody responses compared to mice receiving two immunizations of DnaK+GPI. The serum anti-DnaK antibody responses induced following two or three immunizations were in a dose-dependent manner. Immunization with DnaK alone induced minimal serum anti-DnaK antibody responses (data not shown). Taken together, these results demonstrate that a regimen consisting of three immunizations with DnaK+GPI was the most effective in inducing mucosal and systemic anti-DnaK antibody responses. Furthermore, GPI potentiated IgG1 and IgG2c anti-DnaK responses, suggesting the induction of a mixed type 2 and type 1 response, respectively (Figure 2B).

### Immunogenicity of Tul4

**Figure 3:**
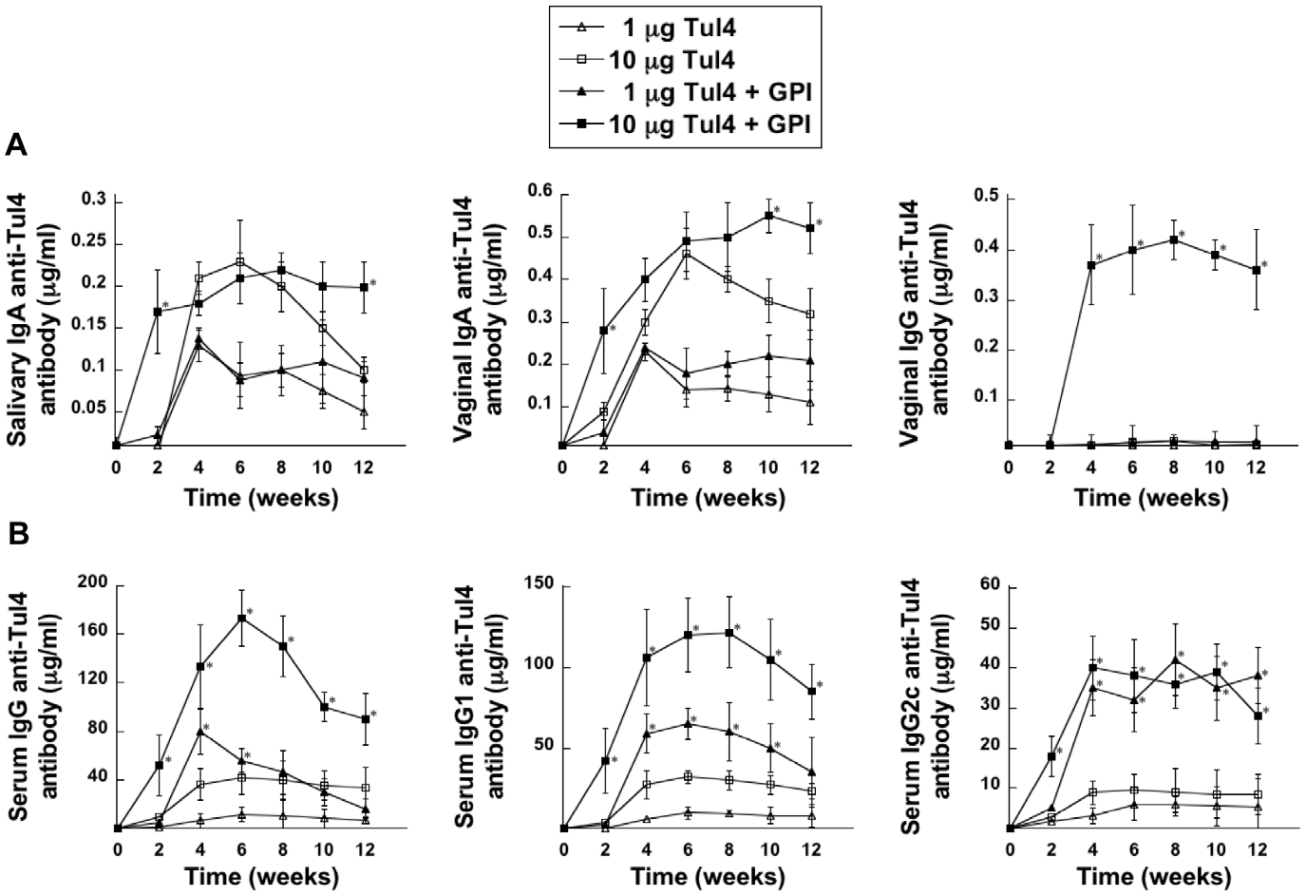
Tul4-specific mucosal and serum antibody responses following i.n. immunization with Tul4+GPI. Mice were immunized via the i.n. route with Tul4 (1 or 10 µg) with or without GPI (100 µg) on days 0, 14 and 28. Individual saliva, vaginal wash and serum samples were collected prior to and at approximately 2-week intervals following immunization. Levels of Tul4-specific IgA in the saliva and vaginal wash samples and IgG in the vaginal wash samples (A), and of IgG, IgG1 and IgG2c in the serum (B), were determined by ELISA. There were 6 mice per experimental group and values are expressed as the mean ± SEM. Significant differences were seen at *P*<0.001 (*) compared with mice immunized with equivalent doses of Tul4 only.

We next determined the immunogenicity of Tul4 and the ability of GPI to modulate the antibody response to this antigen in the mucosal and systemic compartments. Based on our findings with DnaK, we followed the three immunization regimen for our studies with Tul4. Therefore, groups of mice were immunized via the i.n. route with Tul4 alone (1 or 10 µg) or with GPI (100 µg) on days 0, 14 and 28. Unlike DnaK, Tul4 alone induced salivary and vaginal IgA anti-Tul4 antibody responses (Figure 3A), indicating that Tul4 is highly immunogenic. This effect is not likely due to endotoxin contamination, because the bacterial strain used to express recombinant Tul4 is a lipid A mutant of *E. coli* BL21 with a strongly reduced endotoxic activity [39]. Moreover, purified Tul4 was not able to activate macrophages derived from Toll-like receptor 2 (TLR2) knockout (KO) mice, whereas the responses of TLR4 KO and wild type macrophages were comparable (data not shown). In addition, no endotoxin activity was detected in the Tul4 preparation using a Limulus Amebocyte Lysate assay (Cambrex Bio Science, Walkersville, Inc., Walkersville, MD) (data not shown). A dose dependent increase in Tul4-specific salivary and vaginal IgA levels was seen when the immunizing dose was increased from 1 µg to 10 µg, which peaked 4–6 weeks after the initial immunization. Although mucosal IgA anti-Tul4 responses were induced in immunized mice, no Tul4-specific vaginal IgG response was detected. In contrast to the responses observed in mice immunized with Tul4 alone, immunization of mice with Tul4 (10 µg)+the adjuvant GPI resulted in an earlier induction of salivary and vaginal IgA anti-Tul4 antibodies after the first immunization, and the levels detected were significantly higher than those obtained upon immunization with Tul4 alone (10 µg) (Figure 3A). Furthermore, GPI sustained the salivary and vaginal IgA anti-Tul4 responses throughout the 12 weeks experimental period close to the peak levels first detected at 6 weeks, whereas the mucosal IgA antibody responses progressively decreased after reaching peak levels at ∼week 6 in mice immunized with Tul4 alone (10 µg) (Figure 3A). Interestingly, GPI was absolutely required for the induction of vaginal IgG anti-Tul4 antibodies (Figure 3A). Taken together, these results demonstrate that Tul4 alone is able to induce local (salivary) and distal (vaginal) mucosal IgA anti-Tul4 antibody responses following i.n. immunization, whereas, GPI significantly potentiated the longevity of the mucosal IgA anti-Tul4 response and was essential for the induction of a mucosal IgG anti-Tul4 antibody response.

Co-administration of GPI with Tul4 resulted in a significant augmentation of the serum IgG immune response compared to that seen following immunization with Tul4 alone (Figure 3B). Although immunization with Tul4 alone induced peak IgA antibody responses that were close to that seen with GPI+Tul4, similar results were not observed with the serum IgG anti-Tul4 antibody response, suggesting a differential regulation in the responses induced by Tul4 in the mucosal and systemic compartments. However, GPI significantly increased the magnitude and longevity of the serum IgG anti-Tul4 antibody response (Figure 3B). Assessment of the IgG antibody subclass response revealed IgG1 and IgG2c anti-Tul4 antibodies, suggesting the induction of a mixed type 2 and type 1 response, respectively.

### Immunogenicity of a Subunit Vaccine Consisting of DnaK and Tul4 and the Adjuvant GPI

**Figure 4:**
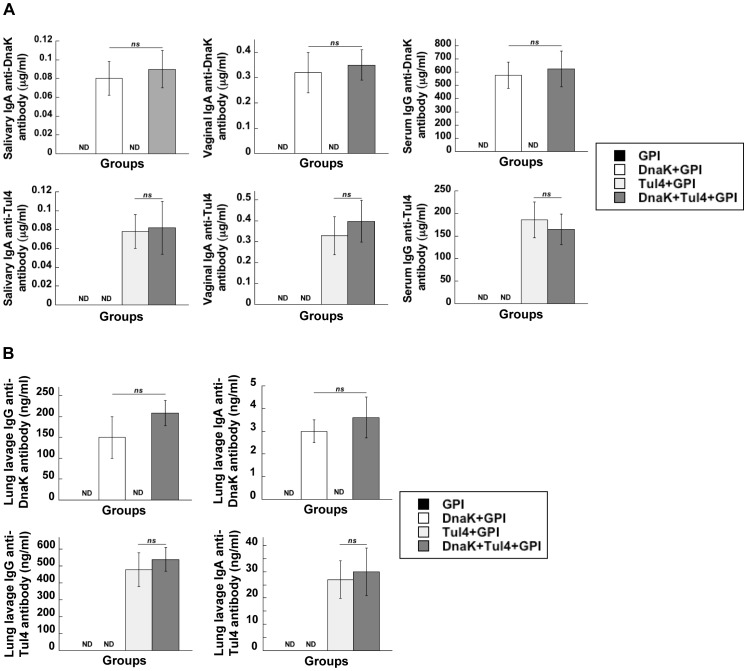
Induction of equivalent DnaK- and Tul4-specific mucosal and serum antibody responses following i.n. immunization with DnaK+Tul4+GPI. Mice were immunized via the i.n. route with DnaK+GPI, Tul4+GPI or DnaK+Tul4+GPI on days 0, 14 and 28. (A) Individual saliva, vaginal wash and serum samples were collected 2 weeks following the last immunization. The levels of DnaK- and Tul4-specific IgA in the saliva and vaginal wash samples and IgG in the serum were determined by ELISA. (B) Individual bronchoalveolar lung lavage samples were collected 2 weeks following the last immunization and the levels of DnaK- and Tul4-specific IgA and IgG were determined by ELISA. There were 4 mice per experimental group and values are expressed as the mean ± SEM. None detected (ND), not significant (*ns*).

We next compared the magnitude of the antigen-specific antibody responses in groups of mice immunized via the i.n. route with DnaK+GPI, Tul4+GPI or with a subunit vaccine preparation consisting of DnaK+Tul4+GPI. The magnitude of the specific mucosal IgA (saliva and vaginal wash) and the serum IgG responses to DnaK and Tul4 were comparable among mice immunized with the subunit vaccine and with DnaK+GPI or Tul4+GPI, respectively (Figure 4A), indicating that the combination of these antigens in a subunit vaccine, does not effect the magnitude of the antibody response induced by each antigen. Since a major interest was to determine the effectiveness of the subunit vaccine in protecting against a respiratory challenge with FT LVS, we next assessed the level of specific antibody activity in lung lavage samples from mice immunized via the i.n. route with the subunit vaccine. IgA and IgG specific antibody activity to DnaK and Tul4 were detected in lung lavages of immunized mice and the levels of the responses were similar to those induced by DnaK+GPI or by Tul4+GPI, respectively (Figure 4B). These results demonstrate that the subunit vaccine induced DnaK and Tul4-specific mucosal and systemic antibody responses that were comparable to immunization with each antigen alone.

### Induction of Antigen-specific CD4^+^ T Cells by Immunization with the Subunit Vaccine

**Figure 5:**
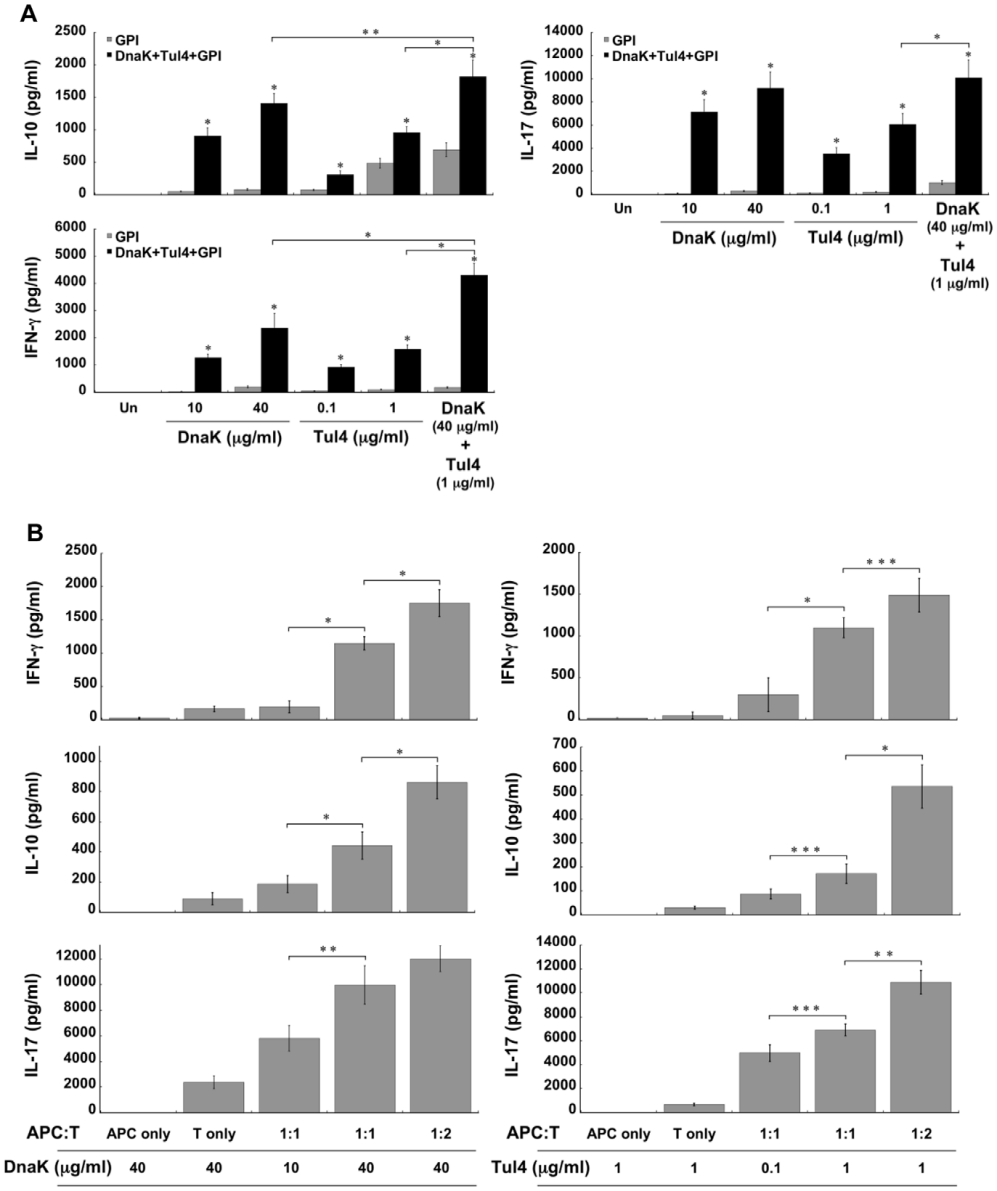
Cytokine production by spleen and purified CD4^+^ T cells from mice immunized with DnaK+Tul4+GPI. Mice were immunized via the i.n. route with DnaK+Tul4+GPI on days 0, 14 and 28. Seven days after the last immunization (A) total splenocytes or (B) purified splenic CD4^+^ T cells co-cultured with irradiated splenocytes/APC at different ratios were stimulated with DnaK or Tul4 at various concentrations, as indicated in figures. Culture supernatants were harvested on day 4 and assessed for the levels of IFN-γ, IL-10 and IL-17 by ELISA. Values are expressed as the mean ± SEM and are representative of 2 separate experiments. Values are significantly different at *P*<0.001 (*), *P*<0.01 (**) and *P*<0.05 (***).

Since FT LVS is an intracellular pathogen, cell-mediated immune responses are crucial for disease resolution [8]. Therefore, we next determined whether the subunit vaccine and the immunization regimen used in these studies activated DnaK- and Tul4-specific cell-mediated immune responses. In vitro stimulation with DnaK or Tul4 of splenocytes derived from mice immunized intranasally with the subunit vaccine, led to IFN-γ, IL-10 and IL-17A production in a dose-dependent manner (Figure 5A). Cell cultures stimulated in vitro with both DnaK and Tul4, showed increased production of IFN-γ, IL-10 and IL-17A compared to the levels obtained from cultures stimulated with each antigen alone (Figure 5A). Minimal or no production of IFN-γ and IL-17A was detected in cultures of spleen cells derived from GPI control immunized mice following in vitro stimulation with DnaK or Tul4, indicating that non-specific activation via GPI did not take place. Interestingly, spleen cells derived from control mice receiving GPI alone produced higher than baseline levels of IL-10 when stimulated in vitro with DnaK, and especially with Tul4. Whether immunization with GPI primed the host for these slight increases in IL-10 upon in vitro stimulation with DnaK or Tul4 is, at this time, not known. However, the significantly higher IL-10 levels in cultures of spleen cells derived from mice immunized with DnaK+Tul4+GPI suggests the antigen-specific nature of this response.

Since CD4^+^ T cells are important for protection against tularemia, we evaluated the induction of antigen-specific CD4^+^ T cells by the subunit vaccine preparation. In vitro stimulation with DnaK or Tul4 of purified CD4^+^ T cells derived from the spleens of mice immunized with the subunit vaccine, induced IFN-γ, IL-10 and IL-17A production in a dose dependent manner (Figure 5B). Furthermore, DnaK and Tul4 induced cytokine production was higher in cultures where the ratio of APC to CD4^+^ T cells was increased from 1∶1 to 1∶2. These results demonstrate that CD4^+^ T cells derived from mice immunized with the subunit vaccine, respond to DnaK and Tul4 in an antigen-specific and dose-dependent manner.

### Effectiveness of the Subunit Vaccine in Inducing a Protective Response Against a Lethal Respiratory Infection with FT LVS

**Figure 6:**
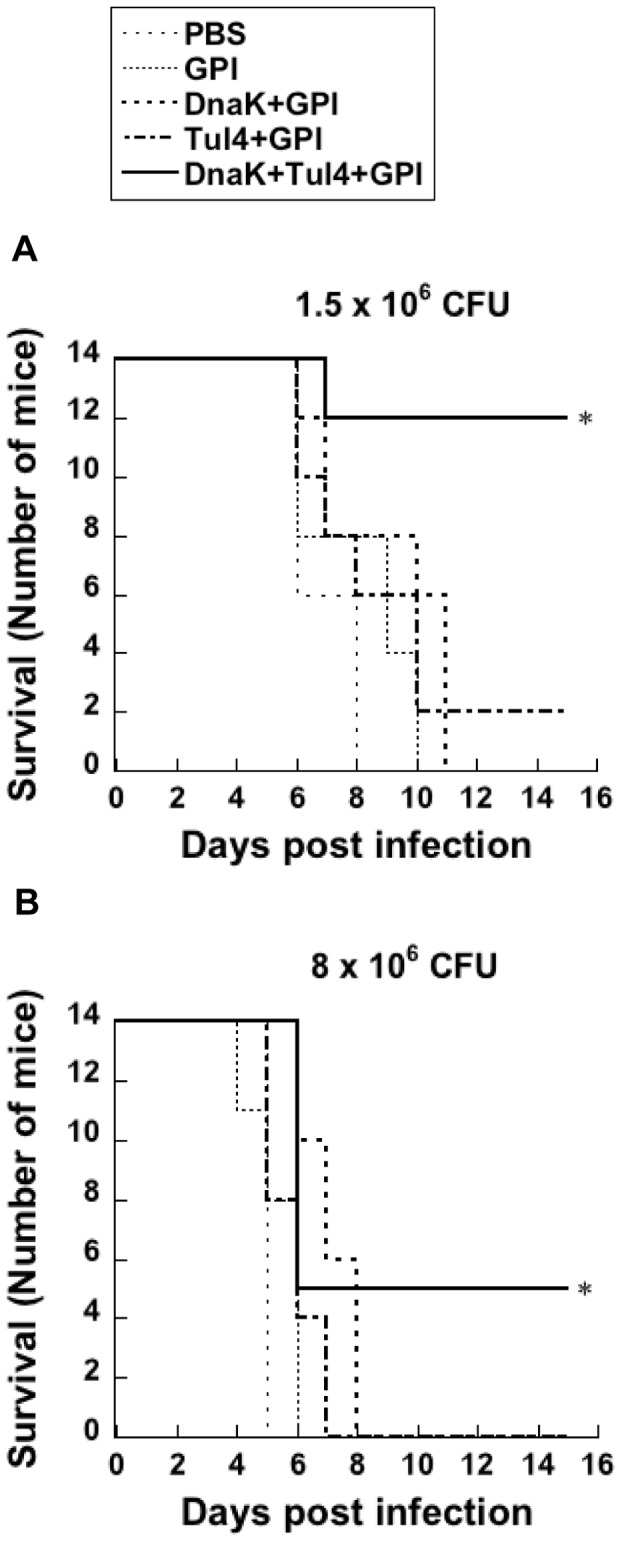
Immune reactivity to DnaK and Tul4 confers protection against a lethal respiratory challenge with FT LVS. Mice were immunized on day 0, 14 and 28 with DnaK+GPI, Tul4+GPI or DnaK+Tul4+GPI. Control groups of mice received PBS or GPI. Two weeks after the last immunization, mice were challenged with either (A) 1.5×10^6^ CFU or (B) 8×10^6^ CFU of FT LVS via the i.n. route and survival was monitored for 30 days. The results show pooled data of two independent experiments (a total of 14 mice per group). Significant differences were seen at *P*<0.001 (*) compared to mice immunized with GPI only.

In order to determine if the immune response induced by the subunit vaccine was protective against a lethal respiratory challenge with FT LVS, groups of immunized and non-immunized mice were challenged via the respiratory route with a lethal dose of FT LVS (1.5×10^6^ CFU) 2 weeks after the third immunization. Non-immunized and GPI immunized control mice succumbed to the infection by day 10 after challenge (Figure 6A). Although mice immunized with GPI and DnaK or Tul4 showed a slightly prolonged survival, they succumbed to the infection by day 13. However, more than 80% of the mice that were immunized with the subunit vaccine were protected against FT LVS challenge (Figure 6A) and survived until the termination of the experimental period.

Next we determined if the observed protection extended to mice challenged with an even higher dose of FT LVS. Immunized and appropriate control groups of mice were challenged with a dose of FT LVS (8×10^6^ CFU) that was approximately 5 fold higher than the dose used in the above described studies. Non-immunized control mice died rapidly, succumbing to the infection by day 6 (Figure 6B). Mice immunized with DnaK or Tul4 and GPI succumbed to the infection by day 8. However, more than 35% of the mice immunized with the subunit vaccine were protected against this highly lethal FT LVS challenge (Figure 6B). Taken together, these results demonstrate the ability of DnaK and Tul4 to provide protection against a lethal dose of FT LVS given via the respiratory route.

### Reduced Bacterial Burden in Tissues of Mice Immunized with the Subunit Vaccine

**Figure 7:**
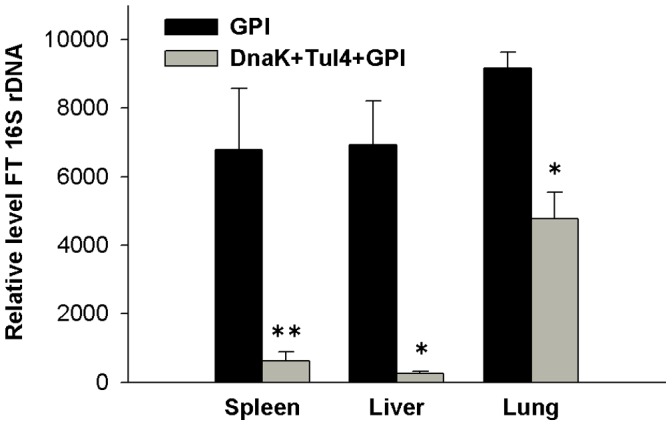
Immunization with subunit vaccine results in reduced bacterial burden. GPI and DnaK+Tul4+GPI immunized mice were challenged with 1.5×10^6^ CFU of FT LVS via the i.n. route. The results show the relative levels of FT LVS-specific 16S rDNA present in the spleen, liver and lungs of infected mice five days after infection. There were 4 mice per group and the values are expressed as the mean ± SEM. Significant differences were seen at *P*<0.01 (*) and *P<*0.05 (**) compared to mice immunized with GPI only.

Mice immunized with the subunit vaccine or with GPI were infected with a lethal dose of FT LVS (1.5×10^6^ CFU) via the respiratory tract, and five days post infection, the spleen, liver and lungs were harvested. Expression of FT LVS-specific 16S rDNA was assessed using real-time PCR as a measurement of bacterial burden in the tissues. Compared to the GPI immunized mice, mice immunized with the subunit vaccine demonstrated significantly reduced levels of FT LVS-specific 16S rDNA in their spleens, livers and lungs (Figure 7).

**Figure 8:**
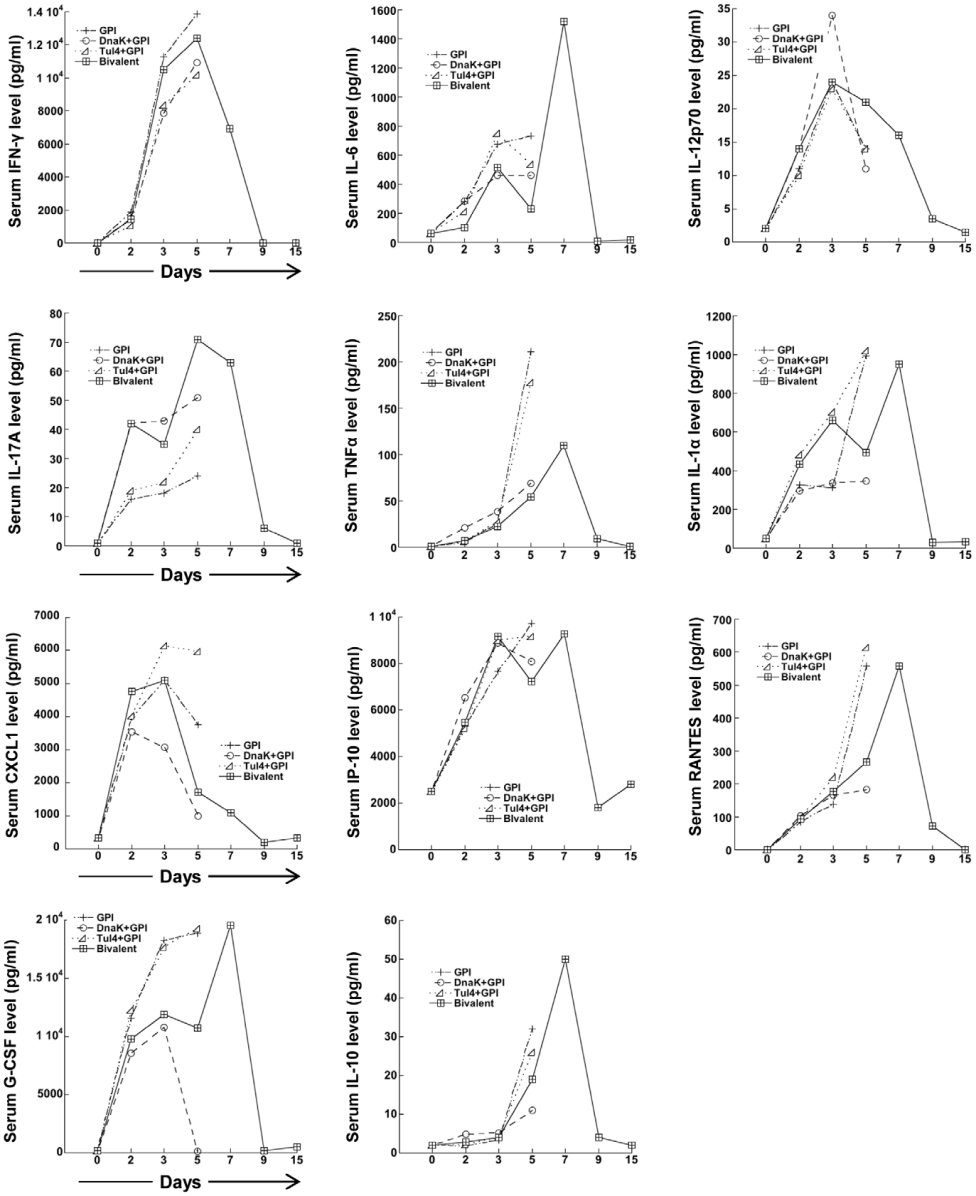
Serum cytokine/chemokine responses in mice immunized with the subunit vaccine and infected with FT LVS via the respiratory route. Levels of various cytokines/chemokines in sera of GPI control mice or of mice immunized with DnaK+GPI, Tul4+GPI, or DnaK+Tul4+GPI and then challenged with FT LVS (1.5×10^6^ CFU) via the i.n. route were assessed using a mouse multiplex kit. Serum samples were collected on days 0, 2 and 3, and then every other day until the mice succumbed to infection or until day 15 from surviving mice. There were 5 mice per group and the values are expressed as the mean.

### Serum Cytokine Patterns of Immunized and Control Mice Infected with FT LVS

Since DnaK and Tul4 exhibit different immunostimulatory activity and both induce a variety of cytokines [30], [31], [40], we reasoned that an interplay between DnaK and Tul4 in the subunit vaccine would result in a cytokine response pattern that correlated with protection and possibly different from that observed with each antigen alone. No difference was seen in the IFN-γ and IP-10 response patterns of infected mice immunized with DnaK or Tul4, or with the subunit vaccine preparation (Figure 8). Interestingly, RANTES, TNF-α and IL-10 levels were similar in all experimental groups by day 3 post infection. By day 5, the levels of these cytokines increased sharply in infected mice immunized with Tul4+GPI or GPI alone, whereas only a slight increase was seen in the sera from mice immunized with DnaK+GPI or with the subunit vaccine (Figure 8). These results suggest that DnaK exerted a downregulatory effect on the production of these inflammatory mediators between days 3 and 5 following infection. Although all experimental groups had comparable G-CSF levels by day 2, by day 3 the response induced in mice immunized with the subunit vaccine was similar to that obtained in DnaK+GPI immunized mice, but not to that observed in mice immunized with Tul4+GPI or GPI alone. The levels of serum IL-6 in infected mice immunized with DnaK+GPI or with DnaK+Tul4+GPI were similar, but lower than that seen in infected mice immunized with Tul4+GPI or GPI alone. IL-6 levels in infected mice immunized with DnaK+GPI remained the same till day 5, whereas a decrease was seen in infected mice immunized with DnaK+Tul4+GPI, in a manner similar to that seen in the sera of mice immunized with Tul4+GPI (Figure 8). These results suggest that Tul4 dampened the induction of IL-6 between days 3 and 5 in infected mice immunized with the DnaK+Tul4+GPI (Figure 8). Interestingly, the levels of G-CSF, RANTES, TNF-α, IL-10 and IL-6 all peaked by day 7 in the surviving mice, but by day 9 the levels dropped to near baseline. In terms of CXCL1, while levels were slightly higher by day 2 in infected mice immunized with DnaK+Tul4+GPI compared to that seen in mice immunized with DnaK or Tul4+GPI; by day 3, levels of this chemokine increased sharply in mice receiving Tul4+GPI, but only slightly in mice receiving DnaK+Tul4+GPI, and in mice immunized with DnaK+GPI, the levels decreased (Figure 8). This finding suggests that Tul4 in the DnaK+Tul4+GPI vaccine preparation exerted some influence on the induction of CXCL1 between 48 and 72 h after infection, yet the sharp decrease in CXCL1 levels by day 5 was perhaps due to an effect by DnaK. The IL-1α response pattern in infected mice immunized with Tul4+GPI or the DnaK+Tul4+GPI vaccine was similar through day 2; however, in infected mice immunized with DnaK+GPI or GPI alone, the level increased only slightly between days 2 and 3 (Figure 8). IL-1α continued to increase sharply through day 5 in mice receiving Tul4+GPI or GPI alone, whereas essentially no increase in IL-1α levels was seen in the DnaK+GPI immunized group. Moreover, a decrease in IL-1α levels between days 3 and 5 was seen in infected mice that had received DnaK+Tul4+GPI (Figure 8), thus approaching the levels seen in infected mice immunized with DnaK+GPI. Noteworthy was the IL-12p70 response in infected mice immunized with DnaK+Tul4+GPI, which followed the pattern induced in mice receiving Tul4+GPI, except that by day 5, the IL-12p70 levels in mice immunized with the combination of GPI and Tul4 or DnaK decreased sharply (Figure 8), while the response of mice immunized with DnaK+Tul4+GPI decreased much slower, reaching baseline levels by day 15 (Figure 8). Lastly, it is interesting that although the pattern of the IL-17A cytokine response was similar in all experimental groups, the level of this cytokine in mice receiving Tul4+GPI were lower than those seen in mice immunized with DnaK+GPI or DnaK+Tul4+GPI (Figure 8). The peak IL-17A response in all groups was observed on day 5; however, the highest levels were seen in mice immunized with DnaK+Tul4+GPI. By the termination of the experimental period, levels were back to baseline. No difference was observed between the serum cytokine/chemokine responses of the control (PBS and GPI) immunized mice, except for IFN-γ, which was slightly higher in the GPI immunized mice on day 5 (data not shown).

## Discussion

Recombinant subunit vaccines are attractive alternatives to inactivated or live attenuated microorganisms, because they can be precisely designed and produced using a standardized manufacturing process, are safer for use in the general population, and their immunogenicity can be potentiated if a proper adjuvant is included in the final formulation. In the present study, a combination of DnaK and Tul4, two distinct immunodominant antigens of FT, together with GPI as an adjuvant, induced antigen-specific mucosal and systemic antibodies and cell mediated immune responses. This immunization regimen protected mice against a respiratory infection with a lethal dose of FT LVS.

Immunization with either DnaK or Tul4 alone did not protect mice against FT LVS infection, yet their combination afforded significant protection, indicating that immune responses to each antigen contributed towards protection. Considering the localization of DnaK (intracellular) and Tul4 (outer membrane), cell mediated, as well as antibody responses could play important roles in protection against FT LVS. Our results show that CD4^+^ T cells from mice immunized with DnaK+Tul4+GPI responded to DnaK and Tul4 stimulation by producing IFN-γ, IL-10 and IL-17A (Figure 5B); and both antigens are processed and presented to CD4^+^ and CD8^+^ T cells upon FT LVS infection (Figure 1). Therefore, we would predict that cell mediated immunity to both antigens plays a role in protection, although further studies are required to address this possibility. Along similar lines, Valentino et al. [32], [41] identified T cell epitopes in DnaK and Tul4 derived from FT, and studies have shown that protection afforded by HSP-based vaccines is primarily dependent on the generation of HSP-specific Th1 responses [42]–[44]. Furthermore, it has become increasingly clear that specific antibodies are important for protection against FT infection [9], [17], [45]–[48]. Tul4, a surface lipoprotein, could be an appropriate antigen for antibody-mediated protection. Recently, using an adenovirus-vectored vaccine, Kaur et al. [23] showed the protective effects of Tul4 against FT LVS infection in mice and implied the role of anti-Tul4 antibodies in protection. In a different study, FopA, another outer membrane protein, was shown to protect mice against FT LVS infection, and antibodies played an important role [21]. Moreover, it is possible that Tul4 may play a role in the adherence of FT to lung cells, and therefore, mucosal IgA anti-Tul4 antibodies may limit bacterial colonization and promote clearance. In this regard, IgA antibodies to FT have been shown to be important for protection against respiratory tularemia [34], [45].

Tul4 is a TLR2 agonist [31], [40]. Therefore, in addition to the adjuvant effects of GPI, it is possible that Tul4 might also act in potentiating innate and/or adaptive immune responses. In this regard, it has been shown that innate immune responses induced via several TLRs can protect against a lethal FT challenge [49]–[51]: however, for optimal protection, TLR agonists need to be administered 1–3 days before infection. Furthermore, since TLRs influence adaptive immune responses [52]; it is possible that Tul4-mediated TLR signaling might provide additional priming for the development of an antigen-specific response. This possibility gains support from the evidence that Tul4 induces a robust antibody response even in the absence of an adjuvant (Figure 3A), and from previous studies demonstrating the importance of TLR2 in host responses to *F. tularensis* [29], [53]–[56].

Cytokines are important players of immune responses, and several cytokines, including TNF-α, IFN-γ, IL-6, IL-12, IL-17A and IL-10, have been shown to be relevant in the host defense against FT infection [8], [57]–[60]. However, our studies did not reveal a specific cytokine pattern associated with protection seen in DnaK+Tul4+GPI immunized mice. In this regard, no difference was observed in the level or pattern of the IFN-γ response among all experimental groups of mice, even though IFN-γ has been shown to be a critical cytokine in host responses against FT infection [58], [61]–[63]. Moreover, between 3–5 days after infection, the levels of IL-12p70, TNF-α and IL-6 were higher in mice immunized with one of the antigens than in mice immunized with both DnaK and Tul4, a pattern similar to that seen with the other cytokines assessed. These findings suggest that in vivo, protection against FT infection likely occurs due to the presence and interactions of numerous inflammatory mediators. Molecules like IP-10, CXCL1, RANTES and G-CSF that were evaluated in the present study are also critical participants in immune responses. For instance, RANTES, IP-10 and CXCL1 (KC), are chemotactic cytokines that belong to the chemokine family and play active roles in recruiting leukocytes to inflammatory sites [64]. Furthermore, G-CSF, besides being a growth factor, is a cytokine that stimulates the survival, proliferation, differentiation and function of neutrophils [65], in addition to its involvement in the regulation of the PI3K/Akt signaling pathway [66]. Finally, the observed changes in cytokine levels between 3 to 5 days after infection are perhaps important for the observed protection, since a differential modulation of the cytokines during this period was observed between infected mice immunized with each antigen and mice immunized with the vaccine preparation containing both antigens.

Five days post i.n. infection of mice with FT LVS, the relative bacterial burden in the livers and spleens was roughly similar to that observed in the lungs of non-immunized mice (Figure 7). This finding indicates that there was a notable systemic dissemination of the bacteria, as has been reported earlier [67]. However, there was a significant decrease in the relative bacterial burden in the lungs, livers and spleens of immunized compared to non-immunized mice. It is interesting that the reduction in bacterial burden was most notable in the liver and spleen. Thus, it is possible that the immunization regimen induced an immune response that prevented a systemic dissemination of the bacteria. Studies by Sharma et al. [68] provide support for this possibility. It is also possible that high titers of antigen-specific IgG, as well as cell-mediated immune responses in the systemic compartment helped control bacterial growth in the spleen and liver. In the case of the lungs, it might be more difficult to clear the infection, especially considering the high number of bacteria that are rapidly lodged in the lungs after i.n. infection [67].

Since the subunit vaccine used in this current study was tested for its efficacy in inducing a protective response against FT LVS and not against the Schu S4 strain, the question of the degree of similarity between FT LVS and Schu S4-derived DnaK and Tul4 needs to be addressed. Analysis of the outer membrane proteins of FT LVS and Schu S4 has revealed almost identical bioinformatics findings and a shared sequence homology of 96 to 100%, which was confirmed with antiserum reactivity to homologous outer membrane proteins, such as Tul4, found in FT LVS and Schu S4 [69]. In a different study, DnaK was among the identified proteins to which T cell hybridomas showed select reactivity upon exposure to FT LVS and Schu S4 lysates [41]. Moreover, the protein sequences encoding the DnaK and Tul4 T cell epitopes are conserved in FT LVS and Schu S4 [32], [41]. Hence, the degree of similarity between the DnaK and Tul4 derived from FT LVS and Schu S4 is extremely high. Based upon these observations, we predict immune reactivity to DnaK and Tul4 in mice immunized with both DnaK+Tul4 will contribute towards a host defense against FT Schu S4 infection. However, it should be kept in mind that the levels of protein expression by a bacterium could influence the efficacy of a vaccine. Studies have shown that the expression of proteins by strains of FT can vary based upon growth conditions, e.g., laboratory media, host macrophages or murine spleens [70], [71]. Therefore, if FT Schu S4 exhibits low levels of DnaK and/or Tul4 expression, especially during the in vivo infection process, this could potentially limit any protective effects of a subunit vaccine. Future studies will be required to investigate this possibility and to determine the effectiveness of our subunit vaccine in protecting against a lethal challenge with FT Schu S4.

Studies have shown that a protective response against FT LVS challenge does not necessarily translate into an effective response against a Schu S4 challenge. For example, immunization with the outer membrane protein FopA was effective against FT LVS, but not a Schu S4 challenge [21]. In another study native outer membrane proteins afforded 50%, but not complete protection against a Schu S4 challenge [22]. Similarly, LPS has been extensively shown to protect mice against FT LVS infection, but a response to LPS alone is not sufficient to completely protect against a type A strain [17], [18], [20], [72]. These findings add to the growing consensus that in order to provide effective protection against more virulent FT subspecies, immune reactivity to several FT antigens will be necessary. Moreover, a combinatorial strategy, where LPS (a T-independent antigen) is chemically conjugated to a protein antigen such as bovine serum albumin, significantly increased protection against a type A strain [18]. This highlights the need to identify a diverse set of FT-specific immunodominant antigens that can stimulate antibody and cell-mediated responses to combat FT infection. Our results support the potential of DnaK and Tul4 as protective antigens against FT LVS infection, and thus add to the limited number, but a growing list of antigens for the development of an effective and safe multivalent vaccine against *Francisella* infection.
